# Psychology as a Natural Science; Applied to Solution of Occult Psychic Phenomena

**Published:** 1889-09

**Authors:** 


### BOOK NOTICES AND REVIEWS.

PSYCHOLOGY AS A NATURAL SCIENCE; APPLIED TO THE SOLUTION OF OCCULT PSYCHIC PHENOMENA. By C. G. Raue, M. D. Philadelphia: Porter & Coates, 1889.

The venerable author of this learned work hardly needs an introduction to *our* readers. He is one of the most eminent of the old guard practicing Homœopathy in Philadelphia—Hering, Lippe, Guernsey, Fellger, who have so nobly sustained the cause of the *only* healing art, and given it its standing in this city. He is the author of the well-known *Pathology and Therapeutic Hints* that has passed through its third edition. He has also published *Raue's*

*Record.* In the preface to this, his latest book, Dr. Raue says: "The application of psychology as a natural science to the solution of occult psychic phenomena implies, first of all, a concise statement and a clear understanding of psychology as a natural science."

Accordingly, on turning over the pages, we find two general ideas pervading them: one of these ideas, as indicated by the title of the book and the fragment of preface quoted, being an explanation of occult phenomena such as mind reading or thought transference, mesmerism, animal magnetism, hallucination, somnambulism, apparitions, haunted houses, and spiritualistic phenomena, the consideration of these occupying the last one hundred and fifty pages.

The other idea being a "concise statement" of the principles of Psychology as a natural science in order that a clear understanding of it may be acquired, and thus enable the student to comprehend the before-mentioned occult phenomena, of which the book is specially designed to treat. This fills the first three hundred and seventy pages, making thus a volume of over five hundred pages.

It would be impossible for us to give a complete analysis of the whole work in the limits of this short article, nor are we competent to do so, owing to lack of time needed for a thorough study of so interesting and able a research. According to our venerable author, the senses, seeing, hearing, smelling, etc., are called *primitive* or original *forces* of the soul. And that he may not be misunderstood, he says at page 14:

"When we speak of primitive forces of the soul we do not mean to imply that they are something separate from the soul, a something possessed or owned by it, but they constitute the very essence or being of which the soul consists at birth."

Page 55:

"They are called *primtive forces* because they are the original and innate powers of the mind, the elements of which the mind consists at birth, and out of which all further capabilities gradually develop."

It will be seen that the author starts out at once by acknowledging the existence of the soul. What is his idea of the soul? We find it on page 441, where he says:

"The human soul is a system of diverse *primitive forces*, from sight and hearing, endowed with the highest capabilities for conscious development down to the lowest in the scale of conscious development—the vital forces."

Again, on page 522:

"The soul consists, on the one hand, of that organized system of immaterial forces, the vital senses, by which it projects itself into the material world. It is composed, therefore, of an immaterial nervous, respiratory circulatory, generative, muscular, bony and cutaneous system; has eyes, ears, nose, mouth, and all the organs in every particular as expressed *materially* in the human body. On the other hand, by its higher immaterial forces, the higher senses, it develops into all those conscious modifications of which we have been treating in this work as cognitions, conations, and feelings and all their wonderful combinations."

All the influences of the external world which excite these *primitive forces* are denominated *stimuli*. When these stimuli excite any of the *primitive forces*, as when the eye is attracted by a beautiful flower, they produce changes in the primitive forces that remain always, though they may not be apparent. These changes are called *vestiges*. The varying degree of vividness in which they are impressed upon the forces constitutes memory with all its variations. Memory, therefore, "consists solely in the quality possessed by the primitive forces of *continuing to persist* in that specific development which has been wrought in them by the action of external stimuli."

Consciousness is defined to be "the repeated action of similar stimuli upon corresponding primitive forces," producing similar *vestiges*. Whenever a sufficient number of similar vestiges have united for us to have a clear consciousness of the object from which the external stimuli were obtained, we say we have a conception of the object.

External *stimuli* have no effect upon the soul unless they are received by free *primitive forces*, which are thereby converted into *vestiges*. If it were possible that the reception of external stimuli (seeing, hearing, etc.) could take place without primitive forces, then it would not be necessary to have sleep in order to renew the primitive forces.

"I" is defined (p. 464) to be "the union in one concept of all perceptions we have made of ourselves. That they all belong to us, to one and the same being is the distinctive feature by which they are united in the one concept "I."

At page 469 we have the explanation why we have the idea that body and soul are one. It is: "Because our body is invariably present to us, and all changes which it undergoes run parallel with our self-consciousness, thus forming by degrees a bond of union so strong that we conceive body and soul as one, or, at least, as linked together seemingly inseparably," whereas the things of the external world are *not* invariably present to our consciousness. In fact, however, our body is as much external to the soul as are any other bodies of the external world.

It will be seen from the foregoing that the five senses of the human body are not mere adjuncts and tools of the soul, but are its integral parts. If, now, we turn to the author's definitions of force and matter we will perceive a similar idea. He denies any separation of the one from the other. At page 343 he begins his argument with the remark: "It is often the case that in speaking of force, the product (motion) is mistaken for force." Then follows an admirably comprehensive exposition of the relationship of heat, light, and electricity, followed by the conclusion:

"Since, furthermore, heat may be transformed into electricity, and electricity into magnetism; since chemical changes may produce electricity or heat or light or magnetism; since gravitation may be transformed to any of these forms, or to all of them in succession, we may safely infer that these so-called *physical forces* are in reality but *modes of motion* caused and originated by the term '*matter*.' Matter, then, instead of being subordinate to these so-called physical forces (which have been thought to play and mold it) is, on the contrary, the very cause. Matter is spent in the origination of these modes of motion, and must, therefore, rightly be considered as the *force* which produces all physical forces.

"We must, then, (in opposition to the common view, which speaks of force and matter as two different things, of which the first uses the latter as the material out of which it molds all existing things), declare that such a distinction is not tenable; that, on the contrary, *every particle of matter is force*, and that the so-called physical forces are but modes of motion produced by these forces. We must, in speaking of matter, discard all notions of *dead* and *inactive*, and fashion our mind to conceive every particle of matter as a force (force constitutes its nature, its essence) which may change its form, but which can never be destroyed."

From this he proceeds to show that the phenomena in plant life do not occur because the plant *possesses* forces, but because the plant "is force and nothing else." He refers to the diffusion of fluids through cells, and the phenomena of assimilation, and reaches a very important point.

We copy his expression: "As force originates motion, it follows that the living tissue contains forces, which forces the dead tissue does not contain. What are these forces? Thus far no one has succeeded in exhibiting them to the senses." Any one familiar with the teachings of the learned physicists and materialists of to-day will appreciate the strong significance of these words. He plainly intimates that these vital forces are a something which can be isolated and presented for examination if only we discover a method.

He tersely asks: "Do they not exist? Did not thallium exist before Mr. Crookes discovered it?"

In man the physical forces in their union constitute the *soul*, while all the material forces in their union constitute the *body*. Man, then, "is a system of interblending material and immaterial forces, and not a juggling together of two diametrically opposite things—spirit and body."

We cannot forbear quoting what is said about the body:

"The ultimate points to which physiological and microscopical anatomical researches have reached, and will ever attain to are the *bioplasts*—microscopical bodies too minute to be weighed, and which appear perfectly structureless, colorless, transparent, and semi-fluid. The smallest of them are spherical and the largest assume the spherical form when free to move in a fluid or semi-fluid medium. There is not one portion of a living, growing tissue the five-hundredth part of an inch in extent, in which bioplasts cannot be demonstrated. They are separated from one another at every period of life, in every part of the body, by a distance little more than the one-thousandth part of an inch. Bioplasts are prior to the cells, the latter being products of the former or material formed. Indeed, all formed material grows out of bioplasts and constitutes the body of the living thing.

"Now, let us suppose we could, by chemical agencies, dissolve all the formed material of the body without destroying its bioplasts—as we can dissolve by hydrochloric acid the calcic elements of any bony structure without destroying its organic constituents—we should then have left a body of such an attenuated form that it would appear as a transparent object, although in its outlines, height, width, depth, and internal arrangement corresponding exactly to the original body, because the bioplasts are not further apart from one another than the one-thousandth part of an inch in any part of the living body. But still it would represent only *material* elements, namely, that portion of the body out of which originally all the formed constituents are evolved."

In selecting the foregoing extracts, our object has been two-fold—first, to show what are the views upon which the author bases his explanations of those strange phenomena that are the puzzle of all mankind—mesmerism, clairvoyance, ghostly visitations, etc.; and, secondly, to show how closely he steers toward the teachings of that singular system of religion or philosophy called Buddhism or Theosophy, which is now spreading over the European and American world with such extraordinary rapidity. The last selection especially will vividly recall to the mind of the Theosophist the Buddhist idea of the astral body—a *fac simile* of the natural body, transparent, yet visible, exactly as described in the quotation.

We have by no means exhausted the rich supply of interesting ideas with which this book is stored. Nor have we even offered to reproduce the explanations of supernatural phenomena, though we are strongly tempted to do so. Suffice it to say that the supernatural appearances are frankly admitted as an undeniable fact, and the explanations are upon the lines of the foregoing quotations, and not founded upon the absurd and dreadful theories of spiritualism.

It is, then, a safe book to read for those who wish to keep their minds free from the nightmare of spiritualism. It adheres strictly to perfectly well-known and accepted scientific principles. It is of absorbing interest and its composition of striking clearness. In these assertions we have not said too much. W. M. J.

